# Inorganic and Polymeric Nanoparticles for Human Viral and Bacterial Infections Prevention and Treatment

**DOI:** 10.3390/nano11010137

**Published:** 2021-01-08

**Authors:** John Jairo Aguilera-Correa, Jaime Esteban, María Vallet-Regí

**Affiliations:** 1 Department of Chemistry in Pharmaceutical Sciences, School of Pharmacy, Research Institute Hospital 12 de Octubre (i+12), Complutense University of Madrid, Plaza Ramón y Cajal s/n, 28040 Madrid, Spain; 2 Clinical Microbiology Department, Jiménez Díaz Foundation Health Research Institute, Autonomous University of Madrid, Av. Reyes Católicos 2, 28040 Madrid, Spain; jestebanmoreno@gmail.com; 3 Networking Research Center on Bioengineering, Biomaterials and Nanomedicine (CIBER-BBN), 28029 Madrid, Spain

**Keywords:** nanomedicine, nanoparticles, viral infection, bacterial infection

## Abstract

Infectious diseases hold third place in the top 10 causes of death worldwide and were responsible for more than 6.7 million deaths in 2016. Nanomedicine is a multidisciplinary field which is based on the application of nanotechnology for medical purposes and can be defined as the use of nanomaterials for diagnosis, monitoring, control, prevention, and treatment of diseases, including infectious diseases. One of the most used nanomaterials in nanomedicine are nanoparticles, particles with a nano-scale size that show highly tunable physical and optical properties, and the capacity to a wide library of compounds. This manuscript is intended to be a comprehensive review of the available recent literature on nanoparticles used for the prevention and treatment of human infectious diseases caused by different viruses, and bacteria from a clinical point of view by basing on original articles which talk about what has been made to date and excluding commercial products, but also by highlighting what has not been still made and some clinical concepts that must be considered for futures nanoparticles-based technologies applications.

## 1. Introduction

Infectious diseases still represent a huge constant threat for humanity [1]. In fact, they hold third place in the top 10 causes of death worldwide and were guilty of more than 6.7 million deaths in 2016 [2]. Among them, 3 million deaths have been caused by lower respiratory infections, 1.4 million deaths have been caused by diarrheal diseases, 1.3 million deaths have been caused by tuberculosis, and one million deaths have been caused by human immunodeficiency virus (HIV) and its complications [2]. These diseases are linked together to both important economic risk for the health system and social ones, and the implications of these associations ranging from individual ordinary people to geopolitical stability [1].

Although this global mortality has recently dropped worldwide thanks to the discovery of antimicrobials and the use of adequate treatments, death rates continue to differ depending on the economy of each country [3]. Despite the existence of many adequate antimicrobial treatments, these will always be connected to the problem of antimicrobial resistance. The introduction in the clinical practice of a new antibiotic is followed by the detection of resistant microorganisms after some variable period in almost all cases. This antimicrobial resistance is due to three major factors: (1) the increasing frequency of antimicrobial-resistant phenotypes among microbes as a result of the selective pressure which the widespread use of antimicrobials exert on the microbes; (2) the globalization, which allows the rapid spread of pathogens from a specific localization to the whole world; and (3) the inappropriate use of antimicrobials in many different settings [4]. The importance of antimicrobial resistance seems to be increasing, and a multidisciplinary, collaborative, regulatory approach is imperiously required for combating this problem, this approach should mainly include rational use of antimicrobials, regulation on over-the-counter availability of antibiotics, improving hand hygiene and improving infection prevention and control, but also the understanding of resistance mechanism and innovation in new drugs and vaccines [5]. One of these innovative approaches is nanomedicine.

**Figure 1:**
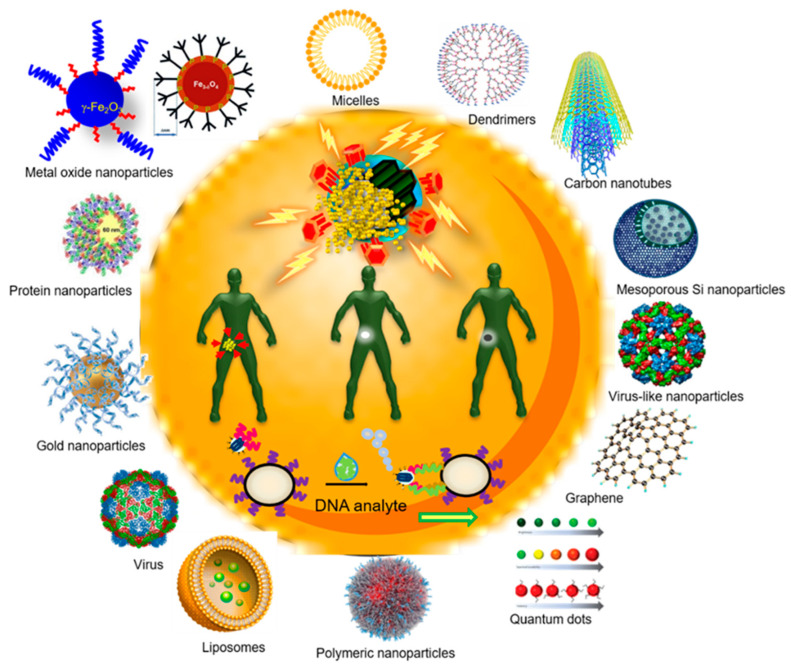
Different types of nanoparticles, both inorganic and polymeric ones, are represented on the outside. Inside, the different functions that can be achieved with the use of nanomedicine are represented: drug release and targeted therapy (improving the pharmacological profile, specific release to target tissues, overcoming biological barriers, and reducing side effects), diagnosis (increased sensitivity, speed and accuracy, early diagnosis, and specific detection of pathological biomarkers), theranosis (focused detection and therapy of diseases, visualizing and evaluating the effectiveness of treatment), and nanodevices (biosensors with greater accuracy and sensitivity, and nanorobots for detection and repair at the cellular level).

Nanomedicine is a multidisciplinary field that is based on the application of nanotechnology for medical purposes and can be defined as the use of nanomaterials for diagnosis, monitoring, control, prevention, and treatment of diseases [6,7,8,9,10,11,12] (Figure 1). Nanomedicine intends to change the clinical practice and introduce novel medicines for both diagnosis and treatment, which can: (1) integrate effective molecules that otherwise could not be used due to their intrinsic high toxicity, (2) exploit multiple mechanisms of action, (3) maximize efficacy whilst dose and toxicity are reduced, and (4) provide drug targeting, controlled and site-specific release [7,11,13]. One of the most used nanomaterials in nanomedicine are nanoparticles, nano-scale particles that show highly tunable physical and optical properties and the capacity to form a wide library of compounds [13]. This manuscript is intended to be a comprehensive review of the available recent literature on nanoparticles used for the prevention and treatment of human infectious diseases caused by different microorganisms from a medical point of view by basing on original articles which talk about what has been made to date and excluding commercial products, but also by highlighting what has not been still made and some clinical concepts that must be considered for futures nanoparticles-based technologies applications.

## 2. Nanoparticles Types and Their Uses

A nanoparticle (NP) is a nanoscopic object with three external nanoscale dimensions [8] generally ranging from nanometers or minus to micrometers [14] which may show antimicrobial properties per se or even act as an antimicrobial carrier. One of the main attractions of NPs is the possibility of functionalization. Functionalization allows altering the chemical and physical properties of an NP with a specific purpose or multiple ones (multifunctionalization). This process can give rise to local or directed antimicrobial delivery, prolong antimicrobial effects, facilitate transport into target microbial cells, and/or locate an area of infection, among others [10,15,16]. This functionalization can be carried out by incorporating different components such as surface ligands that mediate the specific attachment of NPs, linker molecules that release the cargo carried by the NP at the desired site in response to a concrete environmental trigger, one or more therapeutic cargoes that are transported by the NP, and/or a coating, which is generally designed for improving the biocompatibility and bioavailability of the NPs in the inside of human body [16,17].

Considering the temporal order, NPs related to an infection can be divided into NPs from the old toolbox (e.g., liposomes, viruses, and dendrimers), and the young toolbox (e.g., mesoporous silica NPs, carbon nanotubes, fulerenes, graphene). However, the main classification used classifies them into two categories: (1) inorganic and (2) polymeric NPs.

### 2.1. Inorganic Nanoparticles

These NPs include metal and metal oxide NPs which can be synthesized from metals such as gold (Au), silver (Ag), copper (Cu), and/or aluminum (Al) NPs, metal oxides such as iron oxide, magnesium oxide (MgO), titanium oxide (TiO_2_) and zinc oxide (ZnO) NPs, and semiconductors such as silicon and ceramics [18]. Inorganic NPs can withstand harsh process conditions and have been considered safe materials for humans and animals [19]. The antimicrobial effect of these NPs depends on certain characteristics such as size, shape, ζ-potential, ligands, pH, roughness, stability, crystal structure, and material used, but the relationship between these characteristics and their antimicrobial ability is not well-understood to date [20,21]. Their antimicrobial abilities may generally result from at least four mechanisms: reactive oxygen species (ROS) generation, metallic cations release, nanoparticles accumulation on the immediate environment of microbes, and nanoparticle internalization [22]. Furthermore, microbicidal properties from inorganic NPs do not use to select resistance [20,21]. Among inorganic NPs, worthy of note is the mesoporous silica NPs (MSNPs). These nanoparticles are characterized by high chemical stability, high mechanical and heat resistance, and high specific surface area of 1000 m^2^/g. They can be chemically synthesized with particle sizes between 75 and 150 nm, with pore diameters between 2 and 12 nm. Each nanoparticle has about 1400 pores, which provides excellent charge storage inside the nanoparticle to store various molecules, particularly drugs. And they have silanol groups that are key to functionalize both its surface and its interior to suit the specific function being pursued [23]. All these features together back up MSNPs as a more than promising nanocarrier for locally antimicrobial delivery for the treatment of different infections [9,10,17,24,25,26].

### 2.2. Polymeric Nanoparticles

These NPs are made mostly from organic matter. Organic NPs are less stable than inorganic ones, especially at high temperatures and/or pressures [19], but otherwise show excellent biocompatibility, stability, targeting efficiency, and low-hydro-soluble drug storage [27,28]. They represent more than two-thirds of the nano-systems [20,29]. Examples of organic NPs are liposomes, polymeric NPs, micelles, dendrimers, and solid lipid NPs. Liposomes are spherical nanocarriers with a size between 20 and up to 1000 nm [30] which are made from phospholipid bilayer with an aqueous core [28]. Polymeric NPs are colloids solids with a size from 10 to 1000 nm and can be made from polycaprolactone, polyacrylate, but also natural polymers such as alginate, and chitosan or even proteins like albumin [31]. Micelles are also spherical nanocarriers composed of a surfactant monolayer, their size is ranged between 10 and 1000 nm [31]. Dendrimers are symmetrical macromolecules with a sized ranging from10 to 100 nm composed of three parts (a core, a hyper-branched zone, and terminal functional groups) which allows them to charge multiple chemical molecules and to display multiple surface groups [32]. Lipid solid NPs are formulated from lipids which are solid in the physiological temperature and stabilized by emulsifiers and show a size that varies between 10 to 1000 nm [33]. Some advantages of organic NPs such as drug protection against harsh environmental situations, ease of large scale production using high-pressure homogenization technique, biocompatibility, and biodegradability set them up as a better alternative lipid-based system [33]. Nanoemulsions are colloidal dispersions composed by small oil droplets suspended in an aqueous phase whose size varies between 20 to 200 nm [34].

## 3. Nanoparticles and Human Infections

### 3.1. Nanoparticles and Human Viral Infections

Viruses are the most frequent pathogens of epidemic potential. They can give rise to outbreaks in human populations sometimes related to different animal reservoirs [1]. Eleven of the 14 prominent outbreaks humanity have suffered worldwide along 120 years have been viruses, a crucial motive what justifies the importance these pathogens have aroused in nanoparticles field. Moreover, many viruses cause common diseases that can be found worldwide, such as common colds, that can be the cause of enormous economic losses, apart from important morbidity and even mortality [35].

Respiratory viruses are the main cause of mortality worldwide by causing up to 2.7 million deaths in 2015 alone [36]. Among of viruses causing lower respiratory tract infections, the main viruses involved in these diseases are influenza A virus, influenza B virus, metapneumovirus, parainfluenza virus (1–4), rhinovirus, coronavirus (HKU1, NL63, OC43, E229, MERS, SARS-CoV and SARS-CoV-2), enterovirus, and syncytial respiratory virus [37]. Only a few of these viruses, such as influenza A virus, respiratory syncytial virus, and MERS, have been approached by an NPs-based therapy as can be seen in Table 1. Among the most recently published works, the NPs employed against viral infections are mainly used as antimicrobial per se, and then as antimicrobial plus nanocarrier and they are often inorganic and functionalized. The antiviral mechanisms can be grouped into four types, viral deformation/ inactivation, block the viral entry, virus replication inhibition, and cellular apoptosis inhibition. Unfortunately, most of these mechanisms are focused on viral infection prevention, and the works that approach viral treatment are scarce. This point hinders hugely the NPs clinical use against respiratory viral infection because the treatment would be the most useful. Moreover, most of these works are limited to in vitro studies that do not use to evaluate the cytotoxicity on pneumocytes, and the intranasal or inhaled application by using in vivo model should be explored [38]. In vivo models still keep on being an important outstanding issue for this kind of infection. These models must be performed not only for detecting the infecting virus, but also for evaluating the interaction between NPs and the different tissues and organs involving in the respiratory tract (pharynx, larynx, trachea, and lungs) and the possible local inflammation resulting from it.

The contributions which can be added by nanomedicine related to SARS-CoV-2 are noteworthy. This virus is emerging as a huge threat to healthcare and the economy in the whole world [57] and has caused 77,667,963 million cases and at least 1,709,295 [58] million deaths up to date. The main nanotechnological approach can be grouped into three categories: diagnosis technologies, vaccines, and possible therapies [59]. Interestingly, one of these approaches is based on the synthesis of ACE2 coated/embedded nanoflowers or quantum dots for using them to produce chewing gums, nose filters, masks and clothes, and gloves which can inactivate SARS-2 and to limit the viral spread [60].

Viruses are also the most common cause of infectious diarrhea in Western countries [61]. Mortality rate that is inversely proportional to the degree of development of each country [61]. The most common viruses causing diarrhoea are rotavirus, norovirus, and adenovirus. There are few nanoparticles-based prevention or treatment approaches against this type of infection. One of them is Au/CuS core/shell NPs which can inactivate norovirus GI.1 (Norwalk) [62]. Only two vaccine-based approaches have been evaluated: (1) a recombinant rotavirus VP6–ferritin NPs against rotavirus infection [63], and (2) norovirus-rotavirus recombinant polyethylene glycol NPs against these viruses [64]. A recent study asserts that gold spheres of 2–20 nm put onto SiO_2_ spheres or aggregates of 50–200 nm can inhibit adenovirus reproduction by 90–100% in the range of dilutions from 2.5 × 10^−2^ mg/mL to 2.5 × 10^−6^ mg/mL and did not show cytotoxicity in vitro [65]. The potential NPs-based treatment of these diseases is an attractive clinical opportunity that still rises two important issues, (1) the NP-intestine interaction using in vivo model, and (2) the impact of NP on the natural ecosystems and the measures required for minimizing it [66,67] due to this treatment must be ideally orally-administered and, hence, faecally-eliminated.

**Figure 2:**
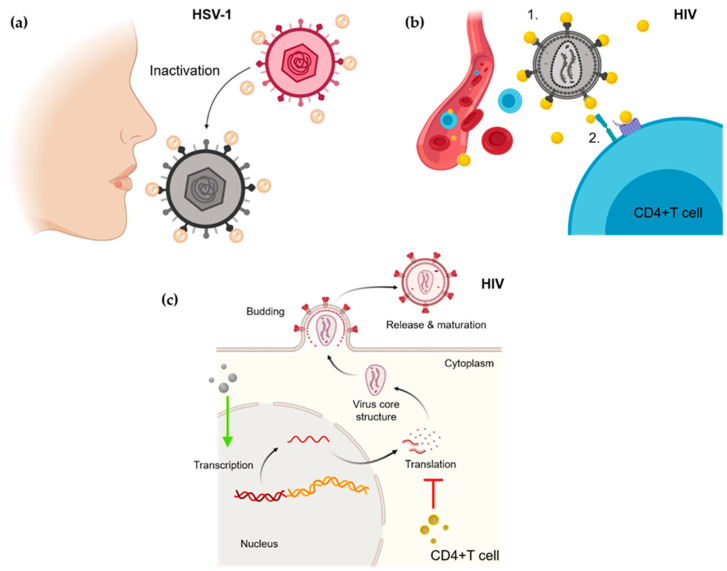
Some of the antiviral mechanisms of NPs. (**a**) Inactivation of HSV-1 virions. (**b**) Inactivation of HIV virions (1) and viral entry blocking (2) throughout interaction between NPs and viral cell receptors. (**c**) Transcription HIV viral DNA favoured by NPs (green arrow) or translation HIV mRNA inhibition (red line).

More than one million sexually transmitted infections (STIs) are diagnosed every day worldwide [68]. Approximately half of these STIs are caused by four viruses which are currently incurable: HIV, herpes simplex virus (HSV), human papillomavirus (HPV), and hepatitis B virus (HBV). New virus as hepatitis C virus have been added to this list due to the new high–risk sexual practices [69]. Some of these NPs-based therapies are summarized in Table 2 and some of them are illustrated in Figure 2. Their currently incurable character up to date of these viruses made more than necessary the exploration of new treatments against them. Not all of these viruses have attracted the same attention from NPs-based therapies, since HIV has taken up most of it. Approximately 38 million (36.2 million were adults, and 1.8 million were children under 15 years of age) people worldwide were infected by HIV in 2019, and it is foreseen that 1.5 million adults and 150,000 children contract the disease each year [70]. Current HIV treatment is based on the use of antivirals that target the various stages in the life cycle of the virus [31]. The current antiretrovirals are nucleoside/nucleotide reverse transcriptase inhibitors, non-nucleoside inhibitors protease inhibitors, entry/fusion inhibitors, CCR5 antagonists, and integrase inhibitors [71]. Despite this treatment cannot cure HIV, do help HIV patients to live longer, healthier lives at the same time which reduces the risk of HIV transmission [72]. Antiretroviral drug resistance threatens to become the main responsible of HIV treatment failure [73]. Therefore, NP-based studies have proposed different alternatives against this virus (Table 2). The main anti-HIV mechanisms based on NP used are viral inactivation, viral entry blocking, cyto-protection, inactivation, infection inhibition, and latency-breaking. From all these mechanisms those that are nearest from the clinical use would be those related to HIV prevention because the NP incorporation to products like lubricants could be locally and topically prevent HIV transmission. However, in vivo model and clinical trials would be necessary for supporting this use. The use of NP as HIV treatment would still remain so far from the clinical application due to the use of CD4+-T cells specific NPs for attacking the virus in its host cells could cause the same damage than the virus in its worst moment, and the use of a non-CD4+-T cells specific NP could represent an inefficient treatment for this infection.

Herpes genitalis can be a result of HSV-1 or HSV-2 infection. This disease can manifest as a primary or recurrent infection, where the virus replicates in epithelial tissue and establishes dormancy in sensory neurons from where it can reactivate periodically as localized recurrent lesions [101]. Due to the pathogeny of this kind of viruses, the best therapeutical approach should be prevention. Several in vitro studies using NPs have demonstrated the ability to inactivate virions, blocking the viral entry, and the viral infection inhibition (Table 2). However, in vivo model and clinical trials would be necessary for backing up this use. At this point, it is worth making a specification between these viruses since HSV-2 infection prevention can take place previously to sexual contact, but HSV-1 infection prevention is much more difficult because this infection can be transmitted by both genital-genital, oral-genital and oral-oral routes, this would hinder the use of specific NPs-based prevention treatment and the application site (mouth or genitals).

Regarding HPV, the nanoparticle-based approaches are mainly focused on the vaccine against this virus and have been recently reviewed [102].

HBV is mainly related to an acute hepatitis that rarely gives rise to fulminant hepatitis. HCB instead does not use to cause acute icteric hepatitis buy do cause a chronic infection in the majority of cases [103]. Both of infections are able to cause cirrhosis and liver cancer ensue in 20% or more over the next 10–50 years [103]. The main mechanism based on NP against viral hepatitis are viral inactivation, viral entry blocking, cyto-protection, inactivation, and infection inhibition (Table 2). Again, the most promising mechanism are associated with the local and topical prevention by incorporating the NP to lubricants. Despite there are multiple functionalizations that can be performed on NPs for becoming them in a perfect medication capturable by the liver (e.g., positively-charged >200 nm-sized nanoparticles [104]), an inflammation derivate from NP-recruitment by liver could generate a hepatitis similar to the viral acute infection.

### 3.2. Nanoparticles and Human Bacterial Infections

Only three of 14 most important outbreaks humanity has suffered worldwide during the past 120 years have been caused by two bacteria, *Vibrio cholerae* and *Yersinia pestis*. Cholera is an acute, secretory diarrhoea provoked by infection with *Vibrio cholerae* of the O1 and O139 serogroups [105]. This disease is endemic in over 50 countries and also causes large epidemics and have been causing severe pandemics since 1812 [105]. Its epidemics have been recently increasing in intensity, duration and frequency, underlying the need for more effective approaches to prevention and control [105]. One of these approaches is the NPs-based therapy. A recent work asserts that ZnO NPs form a complex with cholera toxin, compromises its secondary structure, and blocks its interaction with its receptor in enterocytes and thus reduces cholera toxin uptake [106]. The main inconvenient of this approach would be the possible threat which ZnO NPs would suppose for the environment [107]. Another current work has demonstrated that GM1 ganglioside-coated PLGA hybrid NPs able to recruit cholera toxin and impede its interaction with its receptor in enterocytes by using a murine in vivo model [108]. The biodegradable character of PLGA NPs made them more clinically applicable and harmless to the environment. Plague is caused by *Yersinia pestis* and is infrequent in clinics, though natural plague foci can be found widely distributed around the world [109]. Its three major clinical forms include bubonic, pneumonic, and septicaemic plague and all of them are usually related to a very high mortality rate [109]. Recently, nanomedicine has provided a new promising vaccine based on bacteriophage T4 capsid-derivate nanoparticles which induce immunity by using the capsular protein Caf1 and the low calcium response protein LcrV from *Y. pestis* [110]. Another bacterial species related to outbreaks during human history is *Mycobacterium tuberculosis* [111]. In 2018 only, approximately 10 million incident cases and 1.5 million deaths were attributed to this bacterium [112]. The current increasing existence of multi-drug resistant and extensively drug-resistant strains makes the treatment of this disease an important problem in the present and, potentially, future years [113]. Some current NPs-based approaches have been concerned about this infection and have demonstrated that *M. tuberculosis* show in vitro susceptibility to Ag [114], selenium (Se) [115], and TiO_2_ [116] NPs, but their intracellularly anti-tuberculosis activity remains unclear. Up to date, only a work asserts that PLGA NPs loaded with a highly hydrophobic citral-derived isoniazid analogue promote antibiotic targeting into replicating extra- and intracellular *M. tuberculosis* bacilli [117].

**Figure 3:**
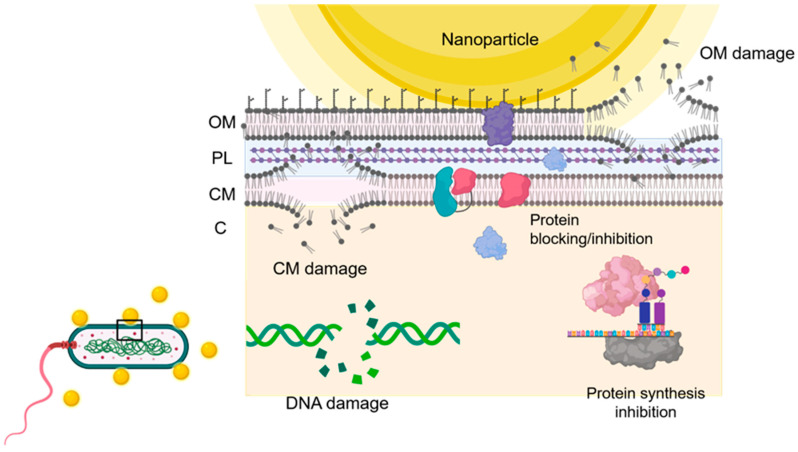
Main antibacterial mechanisms of NPs that show antimicrobial ability per se. OM: outer membrane. PL: peptidoglycan layer. CM: cytoplasmatic membrane. C: cytoplasm.

A present problem with bacterial infections is the emerging threat of antibiotic resistance since bacteria are the most common microorganisms associated with many human infections including most of the healthcare-related infections throughout the world [1]. According to a report from the Centers for Disease Control, more than 2.8 million antibiotic-resistant bacterial infections occur in the United States every year and are associated with more than 35,000 deaths [118]. The priority pathogens related to this antibiotic resistance are (in order of priority) *Acinetrobacter baumannii, Pseudomonas aeruginosa*, enterobacteria (e.g., *Klebsiella pneumoniae* and *Enterobacter cloacae*), *Enterococcus faecium, Staphylococcus aureus, Helicobacter pylory, Campylobacter spp*, *Samonella spp., Neisseria gonorrhoeae, Streptococcus pneumoniae, Haemophilus influenzae*, and *Shigella spp*. [1,119]. Despite this, only a few species from this list have been evaluated in NP-based studies (Table 3). The antibiotic-resistance mechanisms can be summarized in four groups: limiting uptake of an antibiotic, modification of an antibiotic target, inactivation of an antibiotic, and active efflux of an antibiotic [120]. It is indisputable that the antibiotic-resistant bacteria appearance is taking place faster than the new antibiotic discovery and development, a process that requires tremendous economic and labour investment for pharmaceutical industries and is time-consuming [21,121]. Moreover, some of these antibiotic-resistant bacterial infections demand the use of high and/or longer doses of antibiotics or the use of antibiotics generally relegated to a second or even third treatment line because of their toxicity. Thus, behind this scenario, the application of NPs is showed as a potential strategy against these microorganisms [98]. The main antibacterial mechanisms of NPs that show an antibacterial effect per se can be grouped into four categories: (outer and/or cytoplasmatic) membrane damage, protein blocking/inactivation, protein synthesis blocking, and DNA damage (Figure 3). The antibacterial effect of metal NPs have explored and widely reviewed the in vitro antibacterial ability of different heavy metal NPs [122,123,124,125,126,127,128]. Nanoparticles containing Ag [129,130,131,132,133,134,135], Au [136,137,138,139], TiO_2_ [140,141,142], ZnO [143,144,145], CuO [146,147], MgO [148,149], CaO [150,151,152], Al_2_O_3_ [153,154,155], SiO_2_ [156], and clay [157] have shown a great potential antibacterial activity. Some modifications using proteins such as zein protein can give certain metal nanoparticles greater antibacterial capacity, for example zein-coated Au NPs against *P. aeruginosa* [158] or the composite consisting of zein protein and Ag NPs against *S. aureus* [159]. Most of metallic NPs showed positive in vitro antibacterial effects mainly resulting of the bacterial toxic cationic release or ROS generation (Table 3), but these two mechanisms could be diminished by several in vivo considerations. Firstly, the in vivo environment is an polyanionic system [160] where metal cations might be attracted by other host molecules, what might deviate these NPs from its antimicrobial path at systemic level or decrease the cation concentration in the immediate environment of the area of infection. Secondly, ROS generated by NPs could be considerably decreased or neutralized in vivo by biomolecules that can hijack them, e.g., ascorbic acid (vitamin C), uric acid, bilirubin, albumin, glutathione, γ-tocopherol (vitamin E) or ubiquinol of blood lipoproteins [161,162]. Moreover, metals NPs may give rise to immunotoxicity, cytotoxicity, and genotoxicity in both pathogenic bacteria and health human cells [163,164]. Taking into account all these points, the most probable clinical use against bacterial infections of metal NPs would be restricted to topical or local use. Over the last few years, other element such as, nickel [165,166], cerium [166,167], Se [168,169], caesium [170], yttrium [171], palladium [172,173], or superparamagnetic Fe NPs [174] have been recently employed in the battle against antibiotic-resistant bacteria [21,125], but more studies that include cytotoxicity and biocompatibility are necessary.

Liposomes have been considered a useful and valuable tool able to act as drug delivery systems in the treatment of infectious diseases [200]. Nevertheless, a recent and innovative work has demonstrated that liposomes made from cholesterol and/or sphingomyelin were able to sequester the exotoxins of two important pathogenic bacteria, *S. aureus* and *Streptococcus pneumoniae*, and protect from their severe invasive infection in a murine in vivo model [201]. In the same vein, the so-called cell-membrane-coated NP, nanoobjects made from a synthetic NP core which can act as nanocarrier surrounded by a layer of natural cell membrane which mimic the complex biochemical properties of the cells from which they come [202], have been used in a bacterial infection model. In this model, rifampicin-loaded and vancomycin-loaded NPs coated with *S. aureus* extravesicular membrane were able to eliminate macrophage-internalized *S. aureus* and to reduce the bacteremia in a murine in vivo model [203].

STIs provoked by bacterial are gaining importance due to their increasing incidence [1]. Among these bacterial pathogens, there are old known diseases like gonorrhea provoked by *Neisseria gonorrhoeae*, chlamydia caused by *Chlamydia trachomatis*, chancroid provoked by *Haemophilus ducreyi*, granuloma inguinale caused by *Calymmatobacterium granulomatis*, and syphilis provoked by *Treponema pallidum*, but also there are new ones like campylobacter caused by *Campylobacter jejuni* and shigellosis provoked by *Shigella* *sonnei* and *S. flexneri* [204]. Only few of all these diseases have been approached by the NP-based therapy. Most studies have been focused on the treatment of *N. gonorrhoeae* infection. In this sense, Ag NPs plus ceftriaxone [205], mercaptonitrobenzoic acid-coated Ag nanoclusters [206], and chitosan NPs [207] showed a great anti-gonococcal effect with minimal cytotoxicity. It has been also reported that PDGFR-β siRNA-PEI-PLGA-PEG NP significantly reduced the intracellular *C. trachomatis* concentration and bacterial extracellular release from infected cells more than 65% for both of them, while augmenting autophagic degradation and reducing bacterial binding in vitro [208]. Thus, the remaining bacteria STIs set up as a new opportunity for developing new NP-based therapies.

**Figure 4:**
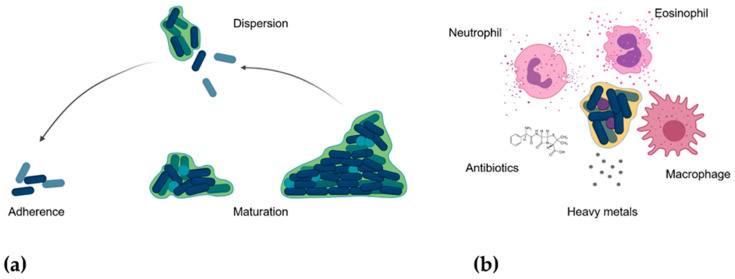
(**a**) Bacterial biofilm formation stages. Green represents the biofilm matrix, and light blue cells represent dead bacteria. (**b**) Inherent characteristics of bacterial biofilm.

On other hand, there is an important idea that has to be considered: bacteria are able to exist in two non-excluding lifestyles: planktonic or free-life form, or in a sessile form named biofilm. A biofilm is a structured bacterial community enclosed in a self-produced polymeric matrix [209] where numerous and complex sociomicrobiological relationships rule [210] (Figure 4a). The adobe-mentioned antibiotic-resistance mechanism can be presented both in planktonic form and in a biofilm of a bacterial strain. However, biofilm form of a bacterium shows different inherent characteristics that give it resistance to almost any unfavourable condition, including the attack of immune system, and antibacterial compounds, such as antibiotics, ROS, and heavy metals [211] (Figure 4b). The biofilm-related infections are estimated about 65% of all bacterial infections [212]. These bacterial infections include both, device-associated and tissue-associated infections [213], and both types have been addressed by NPs-based therapies (Table 3). Some device-related infections are ventricular derivations, contact lenses, endotracheal tubes, central vascular catheters, prosthetic cardiac valves, pacemakers, vascular grafts, tissue fillers, breast implants, peripheral vascular catheters, urinary catheters, orthopaedic implants, and prosthetic joints; and some tissue-related infections are chronic otitis media, chronic sinusitis, chronic tonsilitis, dental plaque, chronic laryngitis, endocarditis, lung infections, kidney stones, biliary tract infections, urinary tract infections, osteomyelitis, and chronic wounds [214], among others. Considering these points, it is pivotal to take into account the NPs susceptibility of biofilm of some bacteria that always trend to form a biofilm, e.g., staphylococci. Still considering the biofilm features, there are NPs-based therapeutical approaches that are a powerful weapon against biofilm-associated infection, for instance, the use of the NPs as an antibacterial or even antibiofilm compounds nanocarrier. It is right here where some porous NPs, e.g., MSNPs, become very important armamentous plethora that can be specifically directed against different types of biofilms according to their functionalization and coatings [24,25,215,216,217]. Recently, this type of nanosystems is becoming established as a future treatment for biofilm-related infection [182,218], e.g., bone infections [27,191,195,196]. Nevertheless, MSNPs applications still arouse questions about their bio-distribution, biocompatibility and the possible inflammatory role they might play during their systemic use [104,219], questions that could be resolved by more and detailed in vivo models.

## 4. Conclusions

Infections continue to represent a great threat for mankind despite recent medical advances. Nanomedicine based on nanoparticles use provides promising new therapies capable of preventing and treating this kind of infections. Here, we summarize and emphasize multiple approaches that employ nanoparticles as therapeutic agents and antimicrobial cargo system against both viral and bacterial infections from a clinical point of view. According to our review, not all of these therapeutic approaches are equally close to being clinically applied, since infection prevention by using nanoparticles is relatively easier to apply than treatment. In addition, not all administration routes are equally applicable, because topical approaches show less obstacles than systemic ones. The way from the bench (in vitro and in vivo studies) to the bed (clinical trials) of nanomedicine is riddled with obstacles. The main challenges linked to the clinical translation of nanoparticles combating viral and bacterial infections are biological issues (e.g., antigenicity, immunotoxicity, and reticular-endothelial system recruitment), safety, biocompatibility, intellectual property, laws and regulations, and cost-effectiveness respect to traditional therapies. These challenges sometimes create an almost insurmountable breach between the nanoparticle and the patient.

## Figures and Tables

**Table 1:** Nanoparticles for therapeutical approaches against respiratory viral infections.

Virus	Nanoparticles (NPs) w/o Conjugate	Size (nm)	Nanoparticle Role	Action Mechanism	Cytotoxicity (%)	Level Study	Cell Lines/Animal Used In Vivo	Reference
Influenza A virus (subtype H1N1)	Ag NPs conjugated with oseltamivir	3	Antimicrobial and nanocarrier	Viral deformation/viral entry blocking/apoptosis inhibition	<10	in vitro	MDCK	[39]
	Ag NPs conjugated with zanamivir	3	Antimicrobial and nanocarrier	Viral deformation/ viral entry blocking/apoptosis inhibition	<20	in vitro	MDCK	[40]
	Au NPs with sialic acid	14	Antimicrobial	Viral entry blocking	<1	in vitro	MDCK	[41]
	Liposome loaded with glycan sialylneolacto-N-tetraose	1–1000	Nanocarrier	Viral entry blocking	-	in vitro/in vivo	MDCK/C57BL/6 mice	[42]
	Se NPs conjugated with zanamivir	82	Antimicrobial and nanocarrier	Viral entry blocking/apoptosis inhibition	<50	in vitro	MDCK	[43]
	Se NPs conjugated with amantadine	70	Antimicrobial and nanocarrier	Viral entry blocking/apoptosis inhibition	<20	in vitro	MDCK	[44]
	Se NPs conjugated with ribavirin	65	Antimicrobial and nanocarrier	Viral entry blocking/apoptosis inhibition	<20	in vitro	MDCK	[45]
	Se NPs conjugated with oseltamivir	100	Antimicrobial and nanocarrier	Viral entry blocking/apoptosis inhibition	<10	in vitro	MDCK	[46]
(H1N1, H3N2, and H9N1 subtypes)	Porous AuNPs	154 ± 37	Antimicrobial	Viral entry blocking	<5	in vitro	MDCK	[47]
(subtypes H1N1, H3N2, PR8, B-Bris, and B-Shan)	Au NPs conjugated with MES	4 ± 1	Antimicrobial	Infection inhibition	-	in vitro	MDCK	[48]
	TiO_2_ NPs conjugated polylysines w/o DNA	>5	Antimicrobial	Virus replication inhibition	-	in vitro	MDCK	[49]
	ZnO NPs conjugated with PEG	18	Antimicrobial	Viral inactivation	<20	in vitro	MDCK	[50]
(H3N2 subtype)	*Ginkgo biloba* leaves polyphenols nanoemulsions	389–988	Nanocarrier	Virucidal and protective effect	0	in vitro	MDCK	[51]
Respiratory syncytial virus	Ag NPs	10	Antimicrobial	Viral replication inhibition	0	in vitro/in vivo	A549, a human alveolar type II-like epithelial cell, and HEp-2/BALB/c mice	[52]
	Ag NPs with curcumin	20	Antimicrobial	Viral entry blocking	<5	in vitro	HEp-2	[53]
	Au NPs conjugated with MES/MUS-OT	2.5 ± 0.7	Antimicrobial	Viral inactivation/viral deformation	0	in vitro/in vivo	HeLa, HEK 293T, CHO-K1, Vero, Hep-2, and HT-1080/BALB/c mice	[54]
	Porous Si NPs	5–50	Antimicrobial	Viral entry blocking	0	in vitro	CEM SS and MA-104	[55]
MERS	Au nanorod conjugated with PH-petide+PEG	54 ± 18	Antimicrobial	Viral entry blocking	0	in vitro/in vivo	Huh-7, 293T, and L02/ICR mice	[56]

Abbreviations: PLA: poly(lactic) acid, RSV: respiratory syncytial virus, MES: 3-mercaptoethylsulfonate, MUS: undecanesulfonic acid, OT: 1-octanethiol, MCDK: Madin-Darby Canine Kidney cells, HeLa: human cervical carcinoma cell line ATCC CCL-2, HEK 293T: human embryonic kidney ATCC CRL-3216, CHO-K1: Chinese hamster ovary cell line ATCC CCL-61, Vero: African green monkey fibroblastoid kidney cells ATCC CCL81, Hep-2: human larynx carcinoma epithelial cell line ATCC CCL-23, and HT-1080: human fibrosarcoma cell line ATCC CCL-121, MERS: Middle East respiratory syndrome.

**Table 2:** Nanoparticles for therapeutical approaches against viral STIs.

Virus	Nanoparticles (NPs) w/o Conjugate	Size (nm)	Nanoparticle Role	Action Mechanism	Cytotoxicity (%)	Level Study	Cell Lines/Animal Used In Vivo	Reference
HIV	PVP-coated Ag NPs	1–10	Antimicrobial	Cyto-protection	-	in vitro	Hut-CCR5	[74]
	Au NPs conjugated with peptide triazoles	13–123	Antimicrobial	Viral inactivation	-	in vitro	HOS.T4.R, 293T	[75]
	Au NPs coated with sulfate-ended ligand	2	Antimicrobial	Infection inhibition	0	in vitro	MT-2	[76]
	Carboxilan dendrimers	-	Antimicrobial	Infection inhibition	<20	in vitro	human CD4+, CD25+, CD127low	[77]
	Carboxilan dendrimers conjugated with RNA decoy	-	Nanocarrier	Cyto-protetion	<20	in vitro	MT4	[78]
	Fe_3_O_4_ NPs loaded with tenofovir+dextran sulphate] + vorinostat	10 ± 3	Nanocarrier	Latency-breaking	0	in vitro	MTT, primary human astrocytes	[79]
	PEG-MA NPs loaded with SMAPP1	-	Nanocarrier	Latency-breaking	-	in vitro	CCRP-CEM	[80]
	PLGA NPs loaded with efavirenz or saquinavir	340	Nanocarrier	Infection inhibition/infection treatment	0	in vitro/ex vivo	TZM-bL, PM-1 and CEMx174/macaque cervicovaginal tissue	[81]
	PLGA NPs loaded with maraviroc, etravirine, and/or raltegravir	200	Nanocarrier	Infection inhibition/infection treatment	<20	in vitro/ex vivo	TZM-bl/human ectocervical explants	[82]
	Porous Si NPs	5–50	Antimicrobial	Viral entry blocking	0	in vitro	CEM SS	[55]
	SiO_2_ NPs conjugated with GPTMS, APTES, and TMPES	354	Nanocarrier	Infection inhibition	0	in vitro	HEK 293T	[83]
HSV-1	Fe3O4 SiO_2_ NPs conjugated with biguanide, polymeric aziridine	150–250	Antimicrobial and nanocarrier	Viral inactivation	<20	in vitro	Vero	
	MES-coated Ag NPs	4	Antimicrobial	Infection inhibition	0	in vitro	Vero	[84]
	PLA NPs loaded with cloroquine	<300	Nanocarrier	Viral entry blocking	<30	in vitro	Vero	[85]
	PLGA nanosphere loaded with acyclovir	190–700	Nanocarrier	Treatment tolerance	-	in vivo	Rabbit	[86]
HSV-2	Liposomes with siRNA	-	Nanocarrier	Infection inhibition	0	in vitro/in vivo	NIH3T3, Vero/BALBc mice	[87]
	PVC NPs loaded with acyclovir	400 ± 6	Nanocarrier	Infection treatment	-	in vivo	Wistar rats	[88,89]
HSV-1/2	Mycosinthetized Ag NPs	4–46	Antimicrobial	Infection inhibition	-	in vitro	Vero	[90]
HBV	Ag NPs	10–50	Antimicrobial	Viral inactivation/Infection inhibition	<50	in vitro	HepAD38	[1]
	(mPEG)–PLA/PEI, mPEG–PLA–chitosan NPs loaded with siRNA	500–800	Antimicrobial and nanocarrier	Inhibition of the HBV surface antigen	<6	in vitro	PLC/PRF/5 c	[91]
	*Ginkgo biloba* leaves polyphenols nanoemulsions	389–988	Nanocarrier	Inhibition of the HBV surface antigen	0	in vitro	HepG 2215	[51]
HCV	PEG-PLDn+PEG-PLEm NPs loaded with antiviral peptides	20–40	Nanocarrier	Cyto-protection	0	in vitro	Huh-7.5	[92]
	Anionic poly(amino acid)-based NPs loaded with antiviral peptides	108	Nanocarrier	Infection treatment	0	in vitro/ in vivo	Huh-7.5/BALBc mice	[93]
	Chitosan-TTP NPs loaded with siRNA	<500	Nanocarrier	Infection treatment	<10	in vitro	CHO K1	[94]
	Solid lipid NPs loaded with RNAi	240	Nanocarrier	Infection treatment	10	in vitro	HepG2	[95]
	Cu NPs	45.4 ± 6.8	Antimicrobial	Viral entry blocking	<5	in vitro	Huh-7.5	[96]
	Dextran-coated magnetic Fe oxide NP conjugated with DNAzyme	75–80	Nanocarrier	Inhibition of expression of the HCV NS3 gene	-	in vitro/in vivo	Huh-7 Luc-Neo/BALBc mice	[97]
	Silibinin-encapsulated liposome	129 ± 3	Nanocarrier	Viral entry blocking/viral inactivation	<20	in vitro	Huh7.5, Huh7.5/Conl/FL-Neo	[98]
	Aptamer-functionalized Fe_2_O_3_ NPs	100	Antimicrobial	Viral inactivation	-	ex vivo	Human plasma	[99]
	Polyanionic carbosilane dendrimers w/o sofosbuvir	-	Nanocarrier	Viral entry blocking	0	in vitro	Huh-7, Huh-7.5.1, Huh-7.5.1-c2	[100]

Abbreviation: PVP: polyvinylpyrrolidine, PLA: polylactide acid, PLG: poly(lactide-co-glycolide, PEG-MA: poly(ethylene glycol) monomethyl ether monomethacrylate, SMSPP: small Molecular Activator of PP1, PGA: polyglycerol adipate, PEG-PLDn: poly(ethylene glycol)-block-poly(α,β-aspartic acid, PEG-PLEm: methoxy-poly(ethylene glycol)-block-poly(L-glutamic acid), TTP: tripolyphosphate.

**Table 3:** Nanoparticles designed for therapeutical approaches against bacterial infections.

Nanoparticles (NPs) w/o Conjugate	Size (nm)	Nanoparticle Role	Action Mechanism	Bacterial Species	Application	Life Style	Cytotoxicity (%)	Level Study	Cell Lines/Animal Used In Vivo	Reference
Au/Ag nanorods	12–14 × 50–55	Antimicrobial	Ag+ release	*E. coli*, MRSA	*S. aureus*-related and *E. coli*-related infections	Planktonic	0	In vitro/in vivo	‒/C57BL6 mice	[175]
TBD-PEG NPs loaded with IR786S, ONOO- and ClO-	50	Nanocarrier	ROS generation	*E. coli*	Wound infection and abscessus cauded by *E. coli*	Biofilm	‒	‒/In vivo	‒/BALBc mice	[176]
Ag NPs anchored on Ti surface	18–24	Antimicrobial	Ag+ release	*S. epidermidis*	Metallic implant infection	Planktonic/biofilm	<10	In vitro/in vivo	MC3T3-E1/Sprague Dawley rats	[177]
ZnO NPs dispersed in a polyvinyl alcohol gel	4–10	Antimicrobial	ROS generation	*E. coli*	Vaginitis	Planktonic	<20	In vitro/in vivo	HepG-2, A-431/ ICR mice	[178]
Ag NPs in a poly (hydroxyethyl methacrylate) gel	‒	Antimicrobial	Ag+ release	*S. aureus/E. coli*	Idwelling implant infection	Planktonic	<20	In vitro/in vivo	NIH-3T3/BALBc mice	[179]
P(GEMADA-co-DMA)-b-PBMA NPs loaded with guanidine lighted with NIR laser	50	Nanocarrier	ROS generation	*S. aureus*	Catheter infection	Planktonic/biofilm	‒	In vitro/in vivo	‒/BALBc mice	[180]
Ag NPs loaded into Ti nanotubes	10–20	Antimicrobial	Ag+ release	MRSA	Metallic implant infection	Biofilm	‒	In vitro/in vivo	MC3T3-E1/Sprague Dawley rats	[181]
Dendrimer-coated MSNPs load with levofloxacin	150	Nanocarrier	Antibiotic-bactericidal effect favored by dendrimer	*E. coli*	*E. coli* biofilm-related infection	Planktonic/biofilm	‒	In vitro	‒	[182]
ε-poly-lysine-coated MSNPs were loaded with histidine kinase autophosphorylation inhibitors (HKAI)	100	Nanocarrier	HKAI-inhibitory effect	*E. coli, Serratia marcensens*	Enterobacterial infection	Planktonic	0	In vitro/in vivo	Caco-2 BBE, RAW 264.7/Zebra fish	[183]
EDC/NHS or ICPTES-functionalized mesoporous SiO_2_ NPs	‒	Nanocarrier	Antibiotic-bactericidal effect of the possible loaded antibiotic	*Francisella tularensis*	Turalemia	Planktonic	‒	In vitro	‒	[184]
Anti-S. aureus antibody-coated Fe3O4/MSNPs loaded with vancomycin	250	Nanocarrier	Vacomycin-bactericidal effect	*S. aureus*	*S. aureus*-associated bacteremia	Planktonic	‒	In vitro	Erytrocyte	[185]
Aptemr-gated MSNPs loaded with vancomycin	177.5	Nanocarrier	Vacomycin-bactericidal effect	*S. aureus/S. epidermidis*	Staphylococcal infections	Planktonic	‒	‒	‒	[186]
Lipidids-coated MSNPs loaded with gentamicin and conjugated with UBI29–41	81.2–99.5	Nanocarrier	Gentamicin-bacteriostatic effect	*S. aureus*	*S. aureus*-associated bone infections	Planktonic/Intracellular	<20	In vitro	MC3T3-E1, RAW 264.7	[187]
Lipidids-coated MSNPs loaded with colistin and conjugated with LL-37	80–99.6	Nanocarrier	Colisitn-bactericidal effect	*P. aeruginosa*	*P. aeruginosa*-associated pulmonar infections	Planktonic/Intracellular	<20	In vitro	A549	[188]
Trehalose-coated PFPA-functionalized MSNPs loaded with isozianid	154-188	Nanocarrier	Isozianid-bactericidal effect	*M. smegmatis*	Mycobacterial infections	Planktonic	‒	‒	‒	[189]
Arginine-coated MSNPs loaded with ciprofloxacin	75	Nanocarrier	Ciprofloxacin-bactericidal effect	*Salmonella enterica* serovar *typhimurium*	Salmonellosis	Planktonic	Low	In vitro/in vivo	RAW 264.7/BALBc mice	[190]
FA-CP-FA-coated MSNPs loaded with ampicilin	80	Nanocarrier	Ampicilin-bactericidal effect	*S. aureus*/*E. coli*	*S. aureus*-related and *E. coli*-related infections	Planktonic	0	In vitro/in vivo	HEK 2931T/Kin Ming mice	[191]
Vancomycin-functionalized mesoporous SiO_2_ NPs	90–127	Nanocarrier	Vacomycin-bactericidal effect	*S. aureus/E. coli*	*S. aureus*-related infections	Planktonic	‒	In vitro/in vivo	‒/BALBc mice	[192]
Concavalin-functionalized MSNPs loaded with levofloxacin	120	Nanocarrier	Levofloxacin-bactericidal effect	*S. aureus*	*S. aureus*-related infections	Planktonic/biofilm	‒	In vitro	‒	[193]
Amino-functionalized MSNPs loaded with levofloxacin	150	Nanocarrier	Levofloxacin-bactericidal effect	*E. coli*	*E. coli*-related infection	Planktonic/biofilm	<10	In vitro	MC3T3-E1	[194]
NB-401 nanoemulsions	400	Nanocarrier	NB-401-bactericidal effect	*P. aeruginosa*, *A. xylosoxidans*, *S. maltophilia*, *Acinetobacter* species, *Pandoraea* species), and *Ralstonia* species	Non-fermenting Gram-negative bacteria-related respiratory infections	Planktonic/biofilm	‒	In vitro	‒	[195]
NB-201 nanoemulsions	350	Nanocarrier	NB-201-bactericidal effect	*P. aeruginosa*	*P. aeruginosa* burn infections	Planktonic/biofilm	‒	In vivo	Sprague-Dawley rats	[196]
N5 and cetylpyridinium chloride nanoemulsions	153	Nanocarrier	Cetylpyridinium-bactericidal effect	*Acinetobacter baumannii*	*Acinetobacter baumannii* infections	Planktonic/biofilm	‒	In vitro	‒	[197]
*Thymus daenensis* oil nanoemulsions	131	Antimicrobial	Oil-bactericidal effect	*Haemophilus influenzae, Pseudomonas aeruginosa*, and *Streptococcus pneumoniae*	Pneumococcal infections	Planctonic	‒	In vitro/in vivo	‒	[198]
*Cleome viscosa* oil nanoemulsions	86	Antimicrobial	Oil-bactericidal effect	Methicillin-resistant *Staphylococcus aureus*, drug-resistant *Streptococcus pyogenes*, and extended spectrum beta-lactamase-producing *Escherichia coli, Klebsiella pneumoniae*, and *Pseudomonas aeruginosa*	Multidrug-resistance bacterial infections	Planctonic	‒	In vitro/in vivo	‒	[199]

Abbreviations: MRSA: methicillin-resistant *S. aureus*. EDC/NHS: 1-ethyl-3-(3-dimethylaminopropyl) carbodiimide/N-hydroxysuccinimide, ICPTES: 3-isocyanatopropyl triethoxysilane, PEPA: perfluorophenylazide, FA: folic acid, CP: calcium phosphate.

## Data Availability

The data presented in this study are available on request from the corresponding author. Some data are not publicly available since some articles are not open access.
